# *“ … we were like tourists in the theatre, the interns assisted almost all procedures … ”* Challenges facing the assistant medical officers training for the performance of caesarean section delivery in Tanzania

**DOI:** 10.1186/s12909-020-02480-z

**Published:** 2021-01-25

**Authors:** Nathanael Sirili, Amani Anaeli, Lilian Mselle, Obadia Nyongole, Siriel Massawe

**Affiliations:** 1 Department of Development Studies, Muhimbili University of Health and Allied Sciences, P.O. Box 65454, Dar es Salaam, Tanzania; 2 Department of Clinical Nursing, Muhimbili University of Health and Allied Sciences, P.O. Box 65001, Dar es Salaam, Tanzania; 3 Directorate of Quality Assurance, Muhimbili University of Health and Allied Sciences, P.O. Box 65001, Dar es Salaam, Tanzania; 4 Department of Obstetrics and Gynaecology, Muhimbili University of Health and Allied Sciences, P.O. Box 65001, Dar es Salaam, Tanzania

**Keywords:** Assistant medical officers, Task sharing, Associate clinicians, Medical education, Tanzania, Primary healthcare

## Abstract

**Background:**

Training of mid-level providers is a task-sharing strategy that has gained popularity in the recent past for addressing the critical shortage of the health workforce. In Tanzania, training of mid-level providers has existed for over five decades; however, concerns exist regarding the quality of mid-level cadres amidst the growing number of medical universities. This study sought to explore the challenges facing the Assistant Medical Officers training for the performance of Caesarean section delivery in Tanzania.

**Methods:**

An exploratory qualitative case study was carried out in four regions to include one rural district in each of the selected regions and two AMO training colleges in Tanzania. A semi-structured interview guide was used to interview 29 key informants from the district hospitals, district management, regional management, AMO training college, and one retired AMO. Also, four focus group discussions were conducted with 35 AMO trainees.

**Results:**

Training of AMOs in Tanzania faces many challenges. The challenges include: use of outdated and static curriculum, inadequate tutors (lack of teaching skills and experience of teaching adults), inadequate teaching infrastructure in the existence of many other trainees, including interns, and limited or lack of scholarships and sponsorship for the AMO trainees.

**Conclusions:**

The findings of this study underscore that the challenges facing AMO training for the performance of Caesarean section delivery have the potential to negatively impact the quality of Caesarean sections performed by this cadre. A holistic approach is needed in addressing these challenges. The solutions should focus on reviewing the curriculum, deploying qualified tutors, and improving the competencies of the available tutors through continuing medical education programmes. Furthermore, the government in collaboration with other stakeholders should work together to address the challenges in teaching infrastructure and providing financial support to this cadre that has continued to be the backbone of primary healthcare in Tanzania. Long-term solutions should consider deploying medical officers at the primary facilities and phasing out the performance of Caesarean section by AMOs.

**Supplementary Information:**

The online version contains supplementary material available at 10.1186/s12909-020-02480-z.

## Background

One of the strategies adopted by many countries globally to lessen the burden of health workforce shortages in the provision of healthcare services is task sharing to mid-level providers [1–3]. Task sharing is the name given to the process whereby less specialized health workers take on some of the responsibilities of more specialized workers in a cost-effective manner without sacrificing the quality of care [4]. To cater for the shortage of health workers, many countries in Africa and other parts of the world have developed Mid-level Health Providers (MLP) in different aspects [5]. The MLP is a health provider that is trained for two to three years after secondary education (lasting around 10 to 12 years), authorized, and regulated to provide healthcare services in an autonomous manner [5]. A good example of MLPs is Associate Clinicians, a cadre that was developed in an effort to curb the shortage of physicians in many countries [2]. Associate clinicians receive different levels of training and carry out different tasks at varying levels and thus labeled differently [1–3]. The World Health Organization (WHO) classifies associate clinicians into two broad categories based on their level of training and scope of practice: associate clinicians, and advanced level associate clinicians [6]. However, despite the vital role played by MLPs in addressing the health workforce crisis, this strategy still suffers many challenges. In some countries, the challenges include: maintaining quality and safety; addressing professional and institutional resistance; sustaining the motivation and performance of other health workers [7, 8].

In Tanzania, the training of MLPs dates back to the 1930s, when the country started to create country-specific cadres to provide services mostly in rural areas (Table 1). These cadres included Clinical Assistants, later named Medical Assistants and in the 2000s renamed Assistant Clinical Officers, Rural Medical Aides (later phased out), Clinical Officers, and many others [9, 10]. In the early 1960s, with the growing population, the critical shortage of medical doctors, the urbanization of medical doctors, and the long-time training required for medical doctors, the country embarked on training a middle-level cadre of clinical practitioners that would perform those roles primarily meant for medical doctors at the district level; these were Assistant Medical Officers, AMOs [10].

**Table 1:** Provision of clinical services at the primary level of health systems in Tanzania

Cadre	Qualification	Duration of training post advanced level secondary education	Level of facility	Responsibility
Assistant Clinical Officer (ACO)	Certificate	Two years	Dispensary	To provide health care for the sick, promote health education, and community mobilisation functions
Clinical Officer (CO)	Diploma	Three years (ACO plus one year)	Dispensary, and Health Centre	To provide health care for the sick, promote health education, and perform administration and community mobilisation functions, particularly for those heading dispensaries
Assistant Medical Officer (AMO)	Advanced Diploma	Five years (CO plus two years)	Health Centre and District Hospital	To provide clinical care to all patients, to perform minor surgeries and emergency obstetric and surgical care at the district level and below. To perform administration and community mobilization functions, particularly for those heading health centres and supervision of lower cadres
Medical Officers	Doctor of Medicine Degree	Five years post advanced secondary education or after CO or AMO education with at least B grade.	Health centre and all level hospitals	To provide clinical care to all patients, to provide obstetric and surgical care. To perform administration and resources mobilization functions, particularly for those heading health centres, hospitals and even districts.

The AMO is an upgraded clinical officer who after working for a minimum of three years undergoes a formal two-year residency training in internal medicine, paediatrics, surgery, obstetrics and gynaecology, and community medicine [10]. The training of AMOs is under the ministry responsible for health and it takes place at an AMO training school located in the selected referral or regional referral hospitals. By 2014, Tanzania had seven AMO schools located in four different zones of Tanzania. Four of these AMO schools are under the private-public partnership between Faith-Based Organizations (FBOs) and the government. Once the two-year residency training is completed, the AMO trainees are awarded an Advanced Diploma in Clinical Medicine. Based on the World Health Organization classification of associate clinicians, AMOs qualify to be advanced level associate clinicians [6]. Therefore, after training, AMOs are expected to provide clinical care, including emergency obstetric and surgical care at the district level and below (Table 1).

A study carried out in the Mwanza and Kigoma regions in Tanzania revealed that over 85% of Caesarean sections and most other obstetric surgeries were performed by AMOs [11]. The situation of the two regions, Mwanza in the lake zone and Kigoma in the western zone, is typical of many regions in Tanzania. Furthermore, by 2012, the country’s health workforce profile revealed that the AMO served a proportionately bigger population than the MD. The AMO population ratio stood at a national average of 1:13,000, with regional variation from 1:13,000 in Dar es Salaam to 1:120,000 in Kagera, while that for MDs was at 1:25,000 with further regional variations [12]. The latter occurs in a country that has less than 50% of the total required health workforce, and less than 40% of the required medical doctors, with only 25% of doctors serving the rural population [13]. Overall, above 70% of the population in Tanzania reside in rural areas [14].

Despite the known contribution of AMOs in addressing the health workforce crisis, anecdotal information reveals that their training succumbs to many challenges that inevitably affect their roles in the task-sharing strategy. Some of the stated challenges are the shortage of tutors, limited sponsorships, and limited career paths. This study, therefore, aimed to explore the challenges facing the assistant medical officers training for the performance of Caesarean section delivery in Tanzania. Specifically, this article aimed to explore the challenges related to training infrastructure, financial support, curriculum, and human resources.

## Methods

An exploratory case study design that adopted a qualitative approach was used for identifying the challenges facing the assistant medical officers training for the performance of Caesarean section delivery in Tanzania. A qualitative case study was necessary to undertake this study, as the training of AMOs is a real phenomenon that involves social processes [15, 16].

### Context of the study

**Fig. 1:**
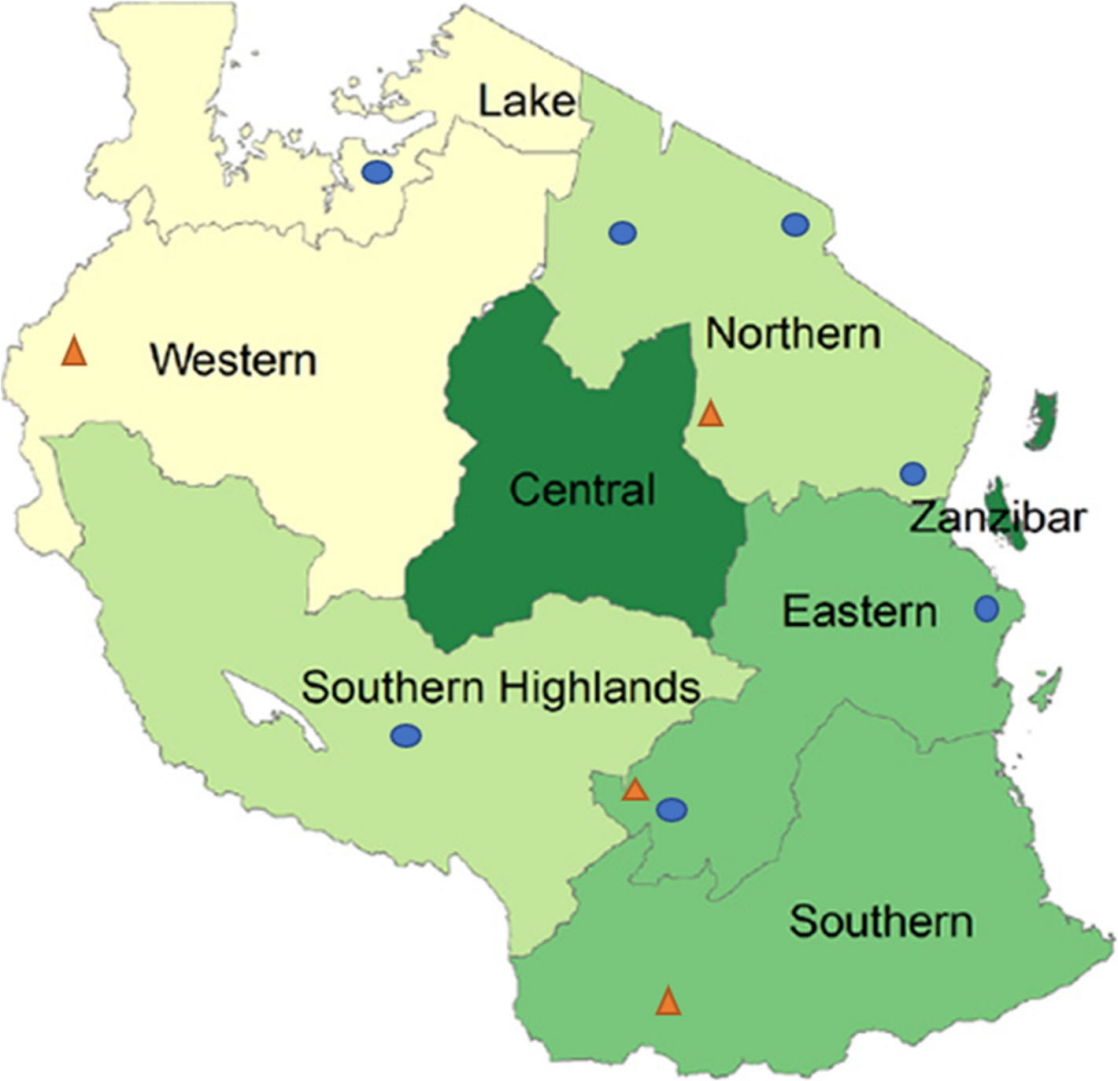
The geography of Tanzania indicating a distribution of AMO training schools and study sites by Zones. Key:  AMO training school  Study sites

Tanzania is divided into seven geopolitical zones, namely: Northern, Eastern, Central, Western, Lake, Southern highlands, and Southern zones. The south, west, and central zones are considered more rural than the rest. The country has seven AMO schools with three located in the Northern zone, two in the Eastern zone, one in the lake zone, and one in the Southern highland zone (Fig 1). Each AMO school had a capacity of admitting up to 40 AMO trainees [10]. Tanzania has five cities: two located in the northern zone, and the rest located in the eastern, lake, and southern highland zones. Dar es Salaam, the largest business city that contains the largest number of the health workforce in the country, is located in the eastern zone.

The provision of healthcare services in Tanzania is organized in a pyramid of three levels: the primary level (comprising district hospital/s, health centres, dispensaries, health posts, and the communities), secondary level (comprising regional and regional referral hospitals), and tertiary level (comprising zonal, specialized hospitals; consultant hospitals; and national hospitals). At all levels, be they rural or urban areas, healthcare services are provided by both public and private health facilities.

This study was carried out in four rural districts (Handeni, Kasulu, Kilombero, and Masasi) located in the four zones (Northern, Western, Eastern and Southern in that order), two AMO schools (one in the northern zone and one in the eastern zone), and at a national level with officials from the ministry of health responsible for the health workforce development and training. The selected AMO schools involved one that was owned and managed by the ministry of health and one under the public-private partnership.

The four zones were purposefully selected to include both rural and urban zones and zones with AMO schools operating under public-private partnership and those operating under the ministry of health (public alone). In each zone, a random selection of rural districts was implemented whereby one rural district was included in the study.

### Study population

This study involved participants from different levels of the healthcare system who are involved in training, supervision of AMOs after training, and those working with the AMOs. These included: principals from AMO training schools, AMOs’ tutors, AMO trainees, Regional Medical Officers, District Medical Officers, Medical Officers in charge of the district hospitals, Senior AMOs at the district hospitals, and one retired AMO (Table 2).

**Table 2:** Study participants (Key Informants and Focused Group Discussants)

Participants	Number	Participants	Number
Regional Medical Officers	04	Principal, AMO schools	02
District Medical Officers	04	AMO tutors	05
Medical Officers in charge	04	Assistant Medical Officers	09
*AMO students (4-FGDs)*	*35*	Retired AMO	01

### Sampling strategy

The purposeful sampling strategy was used to enrol key informants for this study. The enrolment started by identifying the key people who deal with AMOs’ training, supervision, and those who work with AMOs. The latter was implemented through consultation with officials from the directorate of human resources development and training from the Ministry of Health at the section of allied health training and regional and district medical officers from the selected study sites. From the Ministry of Health, the key informants were the officials dealing with overseeing the training of AMOs. These were those dealing with the selection of AMO trainees and overseeing the AMO schools. From the regions and districts, this study involved health managers. In this category, the regional medical officers and district medical officers were included as they are responsible for the work and work environment, permission for further studies, and incentives to the AMOs. At the selected health facilities, this study involved the immediate work supervisors; the medical officers in charge of the district hospitals and senior AMOs at the district hospitals. These are responsible for supervising and overseeing the day- to-day practice of the AMOs, including the performance of Caesarean sections. To get a perspective of changes that have taken place in the training and scope of practice of AMOs, one retired AMO who was trained and practiced as an AMO and later trained as a medical doctor was included in this study. The latter was identified through consultation with senior AMOs from study sites, and a senior gynaecologist who worked with this AMO.

For the focused group discussion, a convenience sampling strategy was used to obtain AMO trainees. Participants who were present during the data collection period and agreed to participate in the study were enrolled from the two AMO schools. In each AMO school, two focused group discussions were conducted, one with male and one with female AMO trainees.

### Data collection

Data for this study were collected between September 2015 and February 2017. Semi-structured interview and focus group discussion guides developed in English and later translated into Kiswahili were used for conducting the Key Informant Interviews (KIIs) and Focused Group Discussions (FGDs). To ensure quality, experienced research assistants who are fluent in both English and Kiswahili were recruited and trained on the objectives of the study, the guides, the informed consent, and the full research process.

Before data collection, the selected informants were contacted by the lead researcher via phone call to set up the appointment for the interview. For the AMO trainees, the principals of the training schools were contacted in advance to organize the FGDs. During data collection, the researchers carried out most of the interviews and FGDs, and the research assistants took field notes. Audio records of the interviews were transferred into a computer by the data manager and kept in a PIN folder in a computer to which he had sole access. The transcripts were all kept by the data manager but only shared with the research team for analysis.

#### Key Informant Interviews

We used different semi-structured interview guides containing questions specific to each group of informants to carry out 29 KIIs. (Table 2). The interview guides were prepared based on experiences of the training of AMOs and task sharing in the country as documented from the available literature [10, 17, 18]. The questions in the guides solicited information on the challenges at the AMO schools, in the districts, and at a national level concerning assistant medical officers training for the performance of Caesarean section delivery in Tanzania. The interviews were carried out at an office designated by the informant and they were recorded using a digital audio recorder. Each interview lasted between 60 and 100 minutes.

#### Focused Group Discussions

We used a semi-structured FGD guide developed based on the competencies detailed in the AMOs’ training curriculum and available literature on task sharing and Caesarean section delivery [9, 10] to carry out four FGDs with AMO trainees from the two AMO schools involved in this study. In each school, we carried two FGDs, one with the female and the other with the male AMO trainees. The number of participants in each FGD ranged from 7-12. In total, 35 AMO trainees participated in the four FGDs. From the FGDs, we explored challenges related to the training of the AMOs for acquiring knowledge and skills for the performance of Caesarean sections as stated in their curriculum. The FGDs lasted between 55 and 120 minutes. A researcher moderated all FGDs.

### Data analysis

All interviews and FGD transcripts were transcribed verbatim. The Kiswahili transcripts were then translated into English before the analysis. A team of four researchers with vast experience in qualitative research, health systems, medical education, and maternal health cross-checked the accuracy and completeness of translations against the original notes before coding. Any gaps identified or clarifications needed were discussed and corrections made accordingly.

Qualitative content analysis as described by Graneheim and Lundman was used to guide the analysis [19]. Codes were extracted from the reduced meaningful unit. Initially, the research team read and reread the transcripts to familiarize themselves with the data before the coding process. The first author developed the initial codebook, based on our study objective and the conceptual understanding of the training of AMOs in Tanzania. The codebook was discussed by all authors, further developed, and a final codebook was imported into NVivo 10 qualitative data analysis computer software. The agreed codebook was tested by independently coding the first two interview transcripts by three authors. Their coding was almost similar and, hence, the codebook was not modified at this time. The team then distributed the transcripts among each other for the coding process.

We coded the meaningful units of text to the codes (nodes) that were found to represent that unit. Some of the meaningful units were coded more than once. At this stage, although the data analysis was guided, it was not confined to the primary codes. Inductive coding was assigned to text segments which represented a new code that was not pre-determined. The new codes were assigned as separate codes or an expansion of the codes available in the initial codebook. All the coded transcripts were then organized by using NVIVO 10 qualitative data analysis software.

Similar codes were grouped together and through abstraction, sub-categories were formed. Through comparison and checking and rechecking of similarities and differences between the sub-categories, the sub-categories were sorted to form categories to reflect the manifest content of the text that were supported with suitable quotes from the transcripts. Further interpretation of the categories was then used to ensure the latent meaning was also brought into focus. The whole process, although described as a linear process, was iterative at all points to ensure that both the manifest and latent meaning of the data is not lost.

### Ethical considerations

Ethical approval was obtained from the Muhimbili University of Health and Allied Sciences Research and Ethical Review Committee. Permission to conduct the study in the four study settings was granted by the Ministry of Health. Written informed consent was obtained from each participant after receiving explanations about the study aim. They were informed that their participation was voluntary and they were free to decline or withdraw at any time in the course of the study. All participants were informed that there was no financial compensation for participating in the study and only water was provided during the interview or discussion. The participants’ privacy was assured by not using their names or facility identity during the data collection and dissemination process through written reports and peer-referred publications. The latter aimed to ensure that no one out of the research team could identify the place where data was collected. Permission was requested for the use of an audio recorder during interviews and discussions.

## Results

From the interviews and FGDs, four categories of challenges facing the assistant medical officers training for the performance of Caesarean section delivery in Tanzania were unveiled. These were: non-responsive static curriculum, limited financial support for AMOs training, human resources inadequacy, and limited teaching infrastructure for AMOs training (Table 3).

**Table 3:** Challenges facing the Assistant Medical Officers training for the performance of Caesarean section delivery in Tanzania

Sub-categories	Categories
▪ Use of an outdated training curriculum ▪ Curriculum focusing more on knowledge rather than combining both knowledge and skills ▪ Curriculum not aligned to the National Education Framework ▪ Curriculum paying little attention to basic sciences ▪ Lack of formal internship after AMO graduation	**Non-responsive static curriculum**
▪ Cutback of financial support from the Ministry of Health ▪ Limited financial support from local governments ▪ Self-financial support as a coping strategy by AMO trainees, burdening the trainees’ families	**Limited financial support for AMOs training**
▪ Shortage of tutors ▪ Use of fresh graduate MDs as tutors ▪ Lack of teaching methodology skills to the tutors	**Inadequacy in human resources**
▪ Shortage of teaching gears (projectors, books, models, etc.) ▪ Limited space for skills training ▪ Existence of many groups of students, including interns, competing for space for practical training ▪ Shortage of office stationers	**Limited teaching infrastructure for AMOs training**

### The use of a non-responsive static curriculum for AMOs training

The use of a static non-responsive curriculum attributed to a lack of regular revision and low emphasis on basic science courses in the curriculum was among the major challenges facing AMOs training in Tanzania.

From the AMOs’ tutors, we found that the curriculum, which is key to the training of AMOs, was written in 2000 (over 15 years ago). This was the first written curriculum since 1963 and it has not been reviewed since 2000. The informants added that the failure to review the curriculum is attributed to the dilemma that surrounds the overall structure of the course. It is worth noting that the AMO program is the only course in Tanzania in which graduates are offered an advanced diploma at the end of the course. In the contemporary academic system of Tanzania, advanced diploma courses have been phased out.

*“ … it has never been reviewed … I know some years back they called us to a review meeting but then came to a conflict with the ministry of education that advanced diploma programmes are no longer in the national academic framework. Since then, I have heard nothing about the review of this curriculum* … ” (KI-AMO training college)

Informants from the district hospitals stated that despite the changes in both training and practice in medicine, AMO training has remained static. They added that the AMO curriculum is experiencing serious knowledge gaps in basic sciences that form the foundation of medical practice. The basic sciences are limited to a period of only eight weeks and they are not the only subjects in that period; rather, they are taught concurrently with clinical rotations.

*“ … in clinical officers training, the training on anatomy and physiology is too basic … it was expected that when one joins AMOs training, then the training on physiology, anatomy, biochemistry and other basic sciences be upgraded … . surprisingly, eight weeks everything is lumped together with other clinical subjects … . then understanding of basic sciences to AMOs is negligible …* ” (KI- Kigoma)

In Tanzania’s health system, there is no formal internship programme after the completion of AMO studies. Analysis of the interviews shows that each council has its own coping mechanism to create an opportunity for “working under the supervision of AMO graduates” before they start to work independently. Depending on the council for which the AMO is working, the time for working under supervision varies from three to twelve months. It is imperative to note that this process is not structured and thus there is no clear-cut goal on what an AMO should gain from this process.

*“ … When they return from their training, we have senior AMOs and MDs here, so we attach the fresh AMOs to seniors in different departments … When the senior is satisfied that the AMO can work independently then s/he moves to another department … the duration varies from three months to 12 months for the individuals … ”* (KI-Mtwara)

### Limited financial support for AMOs training

Across health facilities and colleges, AMO trainees and junior AMOs reported having attempted self-financing as a response to the failure of the government to provide them with financial support. They added that, as government employees, AMO trainees used to receive financial support from the government once they were admitted. However, they reported that financial support was gradually decreasing, and nowadays it remains at the discretion of each council. They added that most councils have failed to provide financial support. The majority of AMO trainees were attempting self-financing, which affects themselves and their families.

*“ … Some council supports their students, and some do not... At your home, you have left children who need school fees … So, it is very difficult to concentrate on a situation when you have no money … sometimes you feel like you have given your family a burden by your decision of coming to school … ” (FGD-AMO training college)*

### Inadequacy in human resources

The challenges of human resources manifested as an absolute shortage of tutors and relative shortage in terms of experienced tutors and lack of pedagogical teaching methods.

Informants from the AMO schools stated that despite the desire to produce a high-quality workforce, AMO schools and the hospitals where AMO are trained face a deficiency of teaching staff. The shortage of teaching staff affects the AMOs training to acquire essential skills, especially in the clinical rotation where the shortage is worse.

*“ … the serious shortage is in clinical rotations, as we do not have a single specialist in this AMO School. … . For instance, in obstetrics and gynaecology we have only one registrar and mostly we rely on the only one available gynaecologist at the hospital who sometimes has traveled for other hospital duties … ” (KI- AMO Training College)*

Furthermore, informants from the AMO training schools stated that most of the tutors were employed by the AMO training schools immediately following their internship without any experience. Given the fact that AMO training is a continuing education that requires proper methods, as most of the students are adults, the informants felt that the use of fresh graduates created an unfavorable learning environment for AMO trainees, who have been exposed to the real workplaces compared to fresh graduate medical doctors. Ascribing to the feelings of the AMOs, the tutors expressed that they also felt uncomfortable teaching clinical skills due to lack of experience.

“ … *Immediately after my internship, I applied for a job through the Ministry of health … . after six months I was posted here as a tutor. At first, it was a very hard job as when I reported I found that most of the tutors were also fresh graduates like me … only four were experienced … for the lectures, it was not a challenge, but for the clinical rotations, yeah it took some time to cope … ”* (KI-AMO training school)

Some tutors who participated in this study added that apart from being medical doctors, they were not equipped with the teaching methodology. Therefore, it was tough for them at the beginning of their work as trainers of adult learners. Trainer and trainee communication in the training session was limited and thus created a communication gap between the two groups. They stated that this made life harder for the AMO trainees, in the long run affecting the quality of the AMOs produced.

*“ … If it is the problem, I think, it is in the methodology; you know teaching is a profession … I mean, that ability to deliver a message to the other person and yet understood … my advice is for evaluation to be done in the given year and the trainers receive training methodology course … ”* (FGD-AMO training college)

### Limited infrastructure for AMOs training

Informants in this study revealed the existence of limited infrastructure, which challenges the delivery of quality training to AMOs. Across AMO schools, a shortage of teaching materials and space for practical training were stated as the main setback. With regards to the teaching materials, overhead projectors, teaching models, computers, skills laboratory, and books were the main outcry of the trainees and trainers. The challenges were reported to exist more at the government-owned schools.

*“ … We used to have enough teaching models but as time goes, they get old, and now we have remained with just a few … We have only two overhead projectors, more than two teachers cannot go to the classes at the same time … we have only one printer and a photocopier, all of them are aged, so it is a challenge during the examinations period. We also do not have a computer in the office so everyone uses a personal laptop if they have one … ”* (KI- AMO training college)

Limited space for practical training was complained of at hospitals by students and junior AMOs across training institutions and districts, limiting them in acquiring the desired competencies. They added that in most AMO schools, there were many other groups of trainees, and the hospitals were small. The latter limited the opportunities for AMO trainees to even observe surgical procedures in the theatre. Some AMOs added that sometimes they were not even included in the schedule for practical training due to a lack of space to accommodate them.

*“ … There are many challenges as I said in the beginning; we were like tourists in the theatre and ward rounds because of the existence of Interns who assisted almost all procedures, Medical students who were also struggling to assist and the residents. In this situation, how do you expect an AMO student to learn? … ”* (KI-Kigoma)

## Discussion

We aimed to explore the challenges facing AMOs training in Tanzania. Our findings have highlighted that although AMOs form the backbone of the district health system and thus the backbone of primary healthcare in Tanzania [10, 20, 21]; and the long history of this cadre of its kind in Eastern, Central, and Southern Africa [10]; AMOs’ training faces multi-dimensional challenges. These challenges are related to the curriculum used in AMOs’ training, financial support to AMO trainees, human resources, and the teaching infrastructure. These challenges altogether threaten the quality of AMO graduates.

The AMOs’ training survived around 40 years without a written curriculum; the latter opens many questions regarding how training was carried out in terms of imparting knowledge, skills, competence, and quality assurance. AMOs perform tasks shared with medical doctors as a way of curbing the critical shortage of doctors in the country [10]. According to the WHO, task sharing should not compromise the quality of services rendered by that group where such tasks are shared [22].

Despite the non-existence of a written curriculum for many decades, our study revealed also that the existing curriculum that was written over 15 years ago has never been revised. This happens in the AMOs’ training while the medical practice is changing rapidly and many medical schools in the country have been regularly revising their training curricula regularly [23, 24]. The existing curriculum has paid little attention to the basic sciences, while they are pivotal in understanding the causation of the disease and why certain decisions must be made regarding treatment, including when and why Caesarean section delivery should occur [25]. The world has transcended rapidly to competency-based education as a way of ensuring that the quality of education is improved by having competent graduates [26, 27]. Therefore, it is high time for the AMOs’ curriculum to be reviewed and changed to a competency-based one as opposed to the knowledge-based one that currently exists. Furthermore, as revealed by our findings, the AMOs’ curriculum the AMOs' curriculum is not in line with the clinical officers' training*.* This makes the understanding of AMOs as upgraded clinical officers shaky. We feel that the training of clinical officers and AMOs needs to be streamlined to make the concept of upgrading a reality. Streaming the curriculum will help to better define the career path for this cadre that has sometimes been referred to as a dead-end career.

Another immediate challenge posed immediately after the training of AMOs at the AMO training schools was the lack of a formal internship program. With the deficiencies in the curriculum and its implementation, an internship program is vital for consolidating the skills and competencies necessary for the performance of Caesarean sections by the AMOs. Unlike Tanzania, some countries have formalized internship programs after graduation of associate clinicians that varies between 6 and 18 months [2].

While training of AMOs focuses on addressing the shortage of the health workforce, our study has revealed that the training of AMOs by itself is affected by the inadequacy of the workforce both in number and skills. This finding reflects what is reported by the Ministry of Health, which indicated that training institutions suffer from a shortage of 74% of the required workforce [28]. The use of inexperienced tutors who are fresh graduates and have not received the teaching methodology course as revealed in this study poses many challenges in the process of imparting knowledge and skills to AMOs. As AMO training is a mature-age entry, as it takes trainees who have pre-service training and clinical experience in many areas, it demands tutors with adequate experience in both teaching and services provision who can give practical examples from the field.

The challenges associated with infrastructure and the existence of multiple trainees and thus limited opportunities for practical training as unveiled by this study bring another dilemma in the AMOs’ training in Tanzania. Although not explicitly the same as our study has documented, studies from different places in Sub-Saharan Africa reveal inadequacies in the training of associate clinicians [8, 29–32]. Limited teaching materials, as revealed in this study, bring in the challenge of quality of graduates. The control of exams, the acquisition of practical skills before touching real patients, and other practical skills are compromised. Not only in Tanzania but in countries where associate clinicians are trained and the question of limited resources is a subject, priority should be given in the provision of the basics in training for quality of care to be guaranteed.

### Methodological consideration

We discuss the methodological consideration of this study in two parts. The first part addresses the trustworthiness of the findings of this study, while the second part addresses the study limitations.

#### Trustworthiness

According to Dahglen and Granheim [19], trustworthiness of a study in a qualitative study is attained when the findings of such a study are worth believing. Lincoln and Guba's Four- Criteria were used to enhance the trustworthiness of the findings of this study [33]; credibility, dependability, transferability, and confirmability [33]. The credibility of the findings of this study was enhanced through the triangulation of informants with experiences and rich information on the study questions. To enhance the credibility and dependability of this study, we used the triangulation of data collection techniques, study settings, and researchers. Data were collected using interview guides and a focus group discussion guide in four different zones with different cultural and socio-economic activities. To confirm that the findings reflected informants’ perspectives rather than the researchers’ understanding of the question under study, categories were inductively generated using content analysis and presented with the support of sub-categories and quotes. The transferability of the findings of this study is enhanced through the description of the study setting, context, data collection process, and analysis.

#### Study limitations

This study is not without some limitations. First, social desirability may have limited the findings of this study, as data collection was led by medical doctors and thus the AMOs may have felt that they should provide desired answers instead of truth value. However, triangulation of methods of data collection, researchers, and sites offset this limitation. Second, only four districts out of 185 districts were involved, with only two AMO training schools. The latter limit generalizability of the findings. However, as this was a qualitative study, the main focus was to get rich information on the AMOs training for the performance of Caesarean section delivery rather than generalization. The latter was enhanced through the triangulation of settings, informants, data collection methods, and researchers. Third and last, this study focused on training of AMOs for the performance of Caesarean section. The latter may have revealed challenges relevant to the training on performance of Caesarean section and left out other pressing challenges on the training of AMOs as a task-sharing strategy. However, this study provides an eye-opener towards studying the challenges facing the training of mid-level providers.

## Conclusions

Training of assistant medical officers for the performance of Caesarean sections as revealed in this study faces many challenges and thus ultimately threatens the quality of the Caesarean services provided by this cadre. For AMOs to reliably perform Caesarean sections, it is high time that revision of its curriculum is aligned to competency-based education. As the AMO training is an in-service programme aimed at improving the performance of an existing cadre, rather than the addition of a new workforce, the provision of financial support by the government or through other development partners needs to be given high consideration to ensure the quality of this training. Employing experienced tutors, the provision of a teaching methodology course to all tutors before they start teaching, and regular refresher’s training is another important strategy that can help resolve the challenges on human resources at the AMO training schools. For infrastructure, efforts should be invested in renovating the existing infrastructure and improving the supply for this cadre to attain the needed competencies.

Finally, while these findings are true for AMOs, we feel that the challenges facing the training of mid-level providers in Tanzania are similar. Therefore, these findings can be used as a starting point to address challenges facing the training of mid-level providers in Tanzania and other settings with a similar context.

## Supplementary Information

**Additional file 1.**

## Data Availability

Transcripts are available. However, sharing is limited for reasons of confidentiality.

## Abbreviations

AMO: Assistant Medical Officer
FGD: Focus Group Discussion
KI: Key Informant
KII: Key Informant Interview
MLP: Mid-level Health Provider
WHO: World Health Organization
