# Supplementary material for: “ … we were like tourists in the theatre, the interns assisted almost all procedures … ” Challenges facing the assistant medical officers training for the performance of caesarean section delivery in Tanzania
Source: BMC Med Educ. 2021 Jan 25;21:72. doi: 10.1186/s12909-020-02480-z (PMC7831162; doi:10.1186/s12909-020-02480-z)
Supplement: Supplementary file 1 — Additional file 1. [file 12909_2020_2480_MOESM1_ESM.doc]

**Appendix: Example of selected guides**

**Interview Guide: Regional Medical Officer (RMO), District Medical Officer (DMO), District Executive Director (DED), Medical Officer Incharge (MOI)**

Completed Consent Form: Yes

*If consent is* ***not*** *obtained, thank them for their time and do not proceed.*

| **Basic Participant Info** |  |
| --- | --- |
| Job Title: | Age: |
| Duration at work place: | Sex: |
| Duration in current position: | Level of Education: |
| Region: | District: |
| Date: | Participant Code: |
| Interviewer name: |  |

**Introduction**: *Task shifting is defined by WHO as the ‘rational’ re-distribution of tasks among health workforce teams. Therefore in order to address the critical shortage of a certain cadre of health workers, other cadres of lower qualification can be trained proficiently to perform such a service. In TZ task shifting is implemented in several health care services. Our focus in this study is on task shifting for cesarean section which is performed by AMOs to address the critical shortage of obstetricians who are quite few in rural areas*

*We appreciate your participation in this study. We have selected you in the study because we believe that being an RMO, you have the authoritative power in the hospital as far as making decisions on health and non health related matters. Therefore you will make a valuable contribution on scaling up of task shifting for CS. By scale up we mean expansion of services to ensure equitable coverage.*

*We would like to reiterate that your participation is voluntary and confidential. Please let me know if you would like to stop the interview at any time.*

**Introductory Questions:** Task shifting for CS by AMOs has been implemented in Tanzania. What are your views regarding its successful scale up?

1. **Focus Area 1 Human Resource management**

*First we’d like to discuss human resource issues related to task shifting for CS.*

1. How has the human resource affected the scale up of task shifting for CS?
   1. How do you rate the number of AMOs being deployed in the Region?
   2. How do you rate the number of AMOs being involved with task shifting for CS?
   3. How do you rate the numbers of other cadres involved with task shifting for CS?
   4. How are AMOs supervised with regards to performance of Cs?
   5. How is the AMOs performance with regards to CS?
   6. How is the performance of other cadres in task shifting for CS?
   7. How are other cadres supervised in task shifting for CS?
   8. What are the lessons learnt in performance of task shifting for CS?
   9. What are the continuing education programs for all cadres involved with task shifting for CS?
2. How has the remuneration package for AMOs affected the scale-up of task shifting for CS?
   1. Do you have recommendations for improving the remuneration package?
3. How does the hospital work to retain cadres of health workers that task shift for CS?
   1. What are some recommendations you would have to improve retention?
4. **Focus Area 4 Infrastructure and Equipments**

*We’d like to discuss about how the infrastructure and equipments affects the scale up of task shifting for CS.*

1. How does infrastructure and equipments facilitate the scale up of task shifting for CS?
   1. What are the lessons learnt if any regarding the available infrastructure and equipments?
2. How does infrastructure and equipments hinder the scale up of task shifting for CS?
   1. What are the challenges?
   2. How best can the challenges be addressed?
   3. What are other special strategies towards ensuring effective and sustainable infrastructure and equipments?

**Closing Question:** After having discussed about task shifting for CS, what most important information would you like to give on how to improve the scale up in Tanzania?

*Thank you for your participation. We look forward to sharing the results of our study with you. If you have any follow-up questions or concerns please contact* ***Prof Siriel Massawe phone number 0754863363***

.

**1.4 Appendix V: Interview Guide: Principal & Tutor AMO school**

Completed Consent Form: Yes

*If consent is* ***not*** *obtained, thank them for their time and do not proceed.*

| **Basic Participant Info** |  |
| --- | --- |
| Job Title: | Age: |
| Duration at work place: | Sex: |
| Duration in current position: | Level of Education: |
| Region: | District: |
| Date: | Participant Code: |
| Interviewer name: |  |

**Introduction**: *Task shifting is defined by WHO as the ‘rational’ re-distribution of tasks among health workforce teams. Therefore in order to address the critical shortage of a certain cadre of health workers, other cadres of lower qualification can be trained proficiently to perform such a service. In TZ task shifting is implemented in several health care services. Our focus in this study is on task shifting for cesarean section which is performed by AMOs to address the critical shortage of obstetricians who are quite few in rural areas*

*We appreciate your participation in this study. We have selected you in our study because as a Principal of AMO school you have valuable knowledge about the training of AMOs in Tanzania. We believe that the training has an impact on implementation and scaling up of task shifting for Cesarean sections by the AMOs in Tanzania. By scale up we mean expansion of services to ensure equitable coverage.*

*We would like to reiterate that your participation is voluntary and confidential. Please let me know if you would like to stop the interview at any time.*

**Introductory Questions:** Task shifting for CS in Tanzania is believed to have had a successful scale up. What are your views and can you explain the reason of your views.

1. **Focus Area 1 AMO Training**

*We shall begin our discussion with issues related to the training of AMO trainees*

1. How has the AMO curriculum facilitated AMOs acquisition of knowledge and skills good attitude to perform CS?
   1. What is the type of curriculum? Does it have clear objectives which can be measurable? Does it enable a student to practice independently after attaining the course ? Does it enable a student to abide to medical ethics and professionalism? Does it enable a student to be evaluated using selected indicators? i.e Competency based?
   2. How has CS been given a special attention in the curriculum?
   3. What is the level of competencies among the trainees in managing obstetric emergency complications?
   4. What is the level of competencies among trainees in diagnosing CS indications?
   5. What is the level of competencies among trainees in performing CS?
   6. How does the teaching and learning environment influence the situation?
   7. What are the supportive supervision programs for AMO trainees in performing CS?
   8. What are post training follow up programs?
   9. What are the lessons learnt, if any?
2. How has the AMO curriculum limited AMOs acquisition of knowledge and skills to perform CS?
   1. Please provide some examples.
   2. What are the challenges?
   3. How could the challenges be better addressed?

**Closing Question:** After having discussed the training and regulation issues , what concluding statement can you give regarding implementation of effective training of AMOs with regards to task shifting for CS?

*Thank you for your participation. We look forward to sharing the results of our study with you. If you have any follow-up questions or concerns please contact* ***Prof Siriel Massawe phone number 0754863363***

**Interview Guide: Practising & Non Practising AMO**

Completed Consent Form: Yes

*If consent is* ***not*** *obtained, thank them for their time and do not proceed.*

| **Basic Participant Info** |  |
| --- | --- |
| Job Title: | Age: |
| Duration at work place: | Sex: |
| Duration in current position: | Level of Education: |
| Region: | District: |
| Date: | Participant Code: |
| Interviewer name: |  |

**Introduction**: *Task shifting is defined by WHO as the ‘rational’ re-distribution of tasks among health workforce teams. Therefore in order to address the critical shortage of a certain cadre of health workers, other cadres of lower qualification can be trained proficiently to perform such a service. In TZ task shifting is implemented in several health care services. Our focus in this study is on task shifting for cesarean section which is performed by AMOs to address the critical shortage of obstetricians who are quite few in rural areas.*

*We have selected you in the study because we believe that being an AMO, you are involved or have been involved in task shifting for CS. Therefore being part of the team in task shifting for CS, you will have valuable information which will guide us in giving appropriate recommendations to enable successful scaling up of task shifting for CS. By Scale up we mean expansion of services to ensure equitable in coverage . We would like to reiterate that your participation is voluntary and confidential. Please let me know if you would like to stop the interview at any time.*

**Introductory Questions:** Task shifting for CS has been implemented in Tanzania. What are your views regarding its successful scale up?

**Focus Area 1 Training:**

*We’d like to begin the discussion on AMO training.*

1. How has the AMO training facilitated you in acquiring knowledge and skills to perform CS?
2. What was your level of competency at graduation (i.e ability to perform independently)?

b. How confident were you in performing CS at graduation?

c. What are the lessons learnt from your AMO training as far as the management of obstetric complications?

d. What are the lessons learnt from your AMO training as far as diagnosing CS indications?

e. What are the lessons learnt from your AMO training as far as performance of CS?

f. How has the AMO training hindered you in acquiring knowledge and skills to perform CS?

g. Please give us examples

h. Are there any skills that are missing/ are weak?

i. What can be done to improve the AMO training?

j. What are the available in service training in emergency obstetric care?

k. What are the in-service programs on emergency obstetric care ?

l. How often do you attend the trainings?

m. How best can such programs be improved?

**Focus Area 2 Human Resource management**

*Having discussed about AMO training, we’d like now to discuss about the human resource issues in relation to scaling up of task shifting for CS****.***

1. Do you still perform CS in your day to day practice?
   1. Could you please give us an explanation if you are not performing the CS
   2. What are the other socio-cultural-economic factors influencing your decision not to perform CS?
   3. For those performing CS, How many CS do you perform in a month and how best can your performance be improved?
2. How have the AMO remuneration package affected the scale –up of task shifting for CS?
   1. Could you please give us an explanation
   2. What are your recommendations to improvement of remuneration package?
3. How have AMO promotion affected the scale up of task shifting for CS?
   1. Could you please explain how often you are being promoted
   2. What are your recommendations to improvement of the AMO promotion scheme?

**Focus Area 3 Skill mix**

*Having discussed about human resource issues, we would now like to discuss about how the skill mix affects task shifting for CS****.***

1. How has the skill mix facilitated the scale up of task shifting for CS?
   1. Could you please explain about the role of each cadre in task shifting for CS?
   2. How efficient does task shifting for CS operate in the hospital setting?
   3. How competent are all cadres in implementation of task shifting for CS?
      1. What are some of your reasons for rating their competencies?
      2. How often do you perform CS
      3. How competent are you in performing CS?
      4. What are your suggestions to improve the competencies?
      5. What are your suggestions to improve other cadres competencies?
   4. What are the lessons learnt in implementation of task shifting for CS in your hospital?
2. How has skill mix hindered the scale up of task shifting for CS?
   1. What are the challenges of skill mix in the scale up of task shifting for CS?
   2. What are your recommendations for improving the skill mix in scaling up of task shifting for CS?

**Focus Area Infrastructure and Equipments**

*Having discussed about the referral system, we would now like to discuss about how the infrastructure and equipments affects task shifting for CS****.***

1. How do equipments and supplies affect scale up of task shifting for CS?
   1. What are your recommendations for the improvement of availability of equipments and supplies?

**Closing Question:** What most important message can you tell us regarding the scale up of task shifting for CS?

*Thank you for your participation. We look forward to sharing the results of our study with you. If you have any follow-up questions or concerns please contact* ***Prof Siriel Massawe phone number 0754863363.***

**1.9 Appendix X: Focus Group Discussion Guide: AMO Trainees**

Completed Consent Form: Yes

*If consent is* ***not*** *obtained, thank them for their time and do not proceed.*

**Demographic Form for Focus Group Discussions**

| Age: |  |
| --- | --- |
| Sex: |  |
| Highest Education Level: |  |
| Training Institution: |  |
| District: |  |
| Current Year of Study: |  |
| Participant Code: |  |
| Date: |  |
| Interviewer Name |  |
| Note taker: |  |

**Introduction**: *Task shifting is defined by WHO as the ‘rational’ re-distribution of tasks among health workforce teams. Therefore in order to address the critical shortage of a certain cadre of health workers, other cadres of lower qualification can be trained proficiently to perform such a service. In TZ task shifting is implemented in several health care services. Our focus in this study is on task shifting for cesarean section which is performed by AMOs to address the critical shortage of obstetricians who are quite few in rural areas.*

*We appreciate your participation in the study. Our study seeks to understand the facilitating factors and hindering factors towards task shifting for CS in Tanzania. We have selected you in the study because part of your training involves proper management of obstetric emergencies, diagnosing obstetric indications for CS as well as performance of CS. We believe that you will provide us with valuable information which will guide us in giving recommendations to improve the scale up of task shifting for CS by AMOs .By scale up we mean expansion of services to ensure equitable coverage. We would like to reiterate that your participation is voluntary and confidential. Please let me know if you would like to stop the interview at any time.*

**Opening Question:** In Tanzania the concept of task shifting has been introduced since many years. One of the areas has been the performance of cesarean section among AMOs and COs in some countries like Malawi. Task shifting has been reported to be successful. What are your views? Please give us the reasons

1. **Training**

*First we’d like to discuss about your training program in relation to acquisition of knowledge and skills towards managing emergency obstetric complications including performance of CS.*

1. How does the AMO training facilitate the AMOs acquisition of knowledge and skills to manage emergency obstetric complications including performance of CS?
   1. What is the type of curriculum? Does it enable one to perform all the clinical duties independently? i.e Competence based? Please give us an explanation
   2. How does the training prepare you to manage obstetric complications effectively?
   3. How does the training prepare you to diagnose maternal indications for CS?
   4. How does the training prepare you perform CS at graduation?
   5. What are the lessons learnt regarding the training in relation to CS performance?
2. How does the AMO training hinder the AMOs acquisition of knowledge and skills to manage emergency obstetric complications including performance of CS?
   1. What are the challenges?
   2. How best can the challenges be addressed?
   3. What are other recommendations which may improve the training with regards to CS performance
3. **Focus Area 2 Infrastructure and Equipments**

*After having discussed on issues of training, we’d now like to discuss on the infrastructure and Equipments to facilitate management of emergency obstetric complications including performance of CS.*

1. How do Infrastructure & equipments facilitate the AMOs acquisition of knowledge and skills to manage emergency obstetric complications including performance of CS?
   1. How do infrastructure & equipments enable you to manage emergency obstetric complications?
   2. How do infrastructure & equipments enable you to diagnose maternal indications for CS?
   3. How do infrastructure & equipments enable you to perform CS?
   4. What are the lessons learnt regarding the availability of infrastructure and equipments?
2. How do infrastructure and equipments hinder the AMOs acquisition of knowledge and skills to manage emergency obstetric complications including performance of CS?
   1. What are the challenges?
   2. How best can the challenges be addressed?
   3. What are other recommendations which may improve the training with regards to CS performance
3. How do Infrastructure & equipments facilitate the scale up of task shifting for CS by AMOs ?
   1. Can you please explain by giving examples
   2. What are the facilitating factors?
   3. What are the lessons learnt?
4. How do Infrastructure & equipments hinder the scale up of task shifting for CS by AMOs
   1. Can you please explain by giving examples
   2. What are the facilitating factors?
   3. What are the lessons learnt?

**Closing Question:** After having discussed about the impact of your training and infrastructure and equipments in relation to task shifting for CS by AMOs, What important messages do you want to tell us regarding task shifting?

*Thank you for your participation. We look forward to sharing the results of our study with you. If you have any follow-up questions or concerns please contact* ***Prof Siriel Massawe phone number 0754863363***

**For Focus Group Facilitator**

| **Focus Group Information** |  |
| --- | --- |
| Training Institution: | Focus Group Code: |
| Date: | Number of participants: |
| Focus Group Discussion Facilitator: | Time started |
|  | Time ended |
